# The Effects of Resistance Training on Physical Function and Quality of Life in Breast Cancer Survivors

**DOI:** 10.3390/healthcare3030695

**Published:** 2015-08-11

**Authors:** Emily Simonavice, Pei-Yang Liu, Jasminka Z. Ilich, Jeong-Su Kim, Bahram H. Arjmandi, Lynn B. Panton

**Affiliations:** 1 School of Health and Human Performance, Georgia College and State University, Campus Box 112, Milledgeville, GA 31061, USA; E-Mail: Emily.simonavice@gcsu.edu; 2 School of Nutrition and Dietetics, The University of AkronSchrank Hall South 210M, Akron, OH 44325, USA; E-Mail: liu4@uakron.edu; 3 Department of Nutrition, Food, and Exercise Sciences, Florida State University, Tallahassee, FL 32306, USA; E-Mails: jilichernst@fsu.edu (J.Z.I.); jkim6@fsu.edu (J.-S.K.); lpanton@fsu.edu (L.B.P.); 4 Center for Advancing Exercise and Nutrition Research on Aging, Florida State University, 0412 Sandals Bldg., Tallahassee, FL 32306, USA; E-Mail: barjmandi@fsu.edu

**Keywords:** breast cancer survivors, physical function, quality of life, resistance training

## Abstract

Breast cancer survivors (BCS) exhibit decreased physical function and quality of life (QOL) following cancer treatments. Resistance training (RT) may elicit positive changes in physical and mental well-being. This study assessed 27 BCS, pre-and post-intervention (six months) on the following variables: muscular strength (via one repetition maximum (1RM) of chest press and leg extension), physical function (via the Continuous Scale-Physical Functional Performance test) and QOL (via the Short Form-36 survey). RT consisted of two days/week of ten exercises including two sets of 8–12 repetitions at 52%–69% of their 1RM. A repeated measures analysis of variance revealed BCS significantly (*p* < 0.05) increased upper (71 ± 22 to 89 ± 22 kg) and lower body (74 ± 18 to 93 ± 24 kg) strength, total physical function (65.5 ± 12.1 to 73.6 ± 12.2 units) and the subcomponents of physical function: upper body strength (63.5 ± 16.3 to 71.2 ± 16.8 units), lower body strength (58.5 ± 14.9 to 68.6 ± 16.3 units), balance and coordination (66.5 ± 12.2 to 74.6 ± 11.6 units), and endurance (67.2 ± 12.0 to 75.0 ± 11.6 units). No changes were observed over time for subjective measures of physical function and QOL. Results showed RT could be an effective means to improve objective physical function in BCS. Further research is needed to clarify the effects of RT on subjective physical function and QOL.

## 1. Introduction

Approximately 1.6 million new cases of cancer were expected to be diagnosed in the United States in 2014. Of these cancer diagnoses, it was estimated that nearly 300,000 were breast cancer [1]. While the prognosis of breast cancer is improving, there is an estimated 2.9 million breast cancer survivors (BCS) left to deal with the numerous adverse side effects caused by the cancer itself and/or the cancer related treatments [2]. Previous studies suggest that BCS encounter an array of detrimental physical changes resulting from the treatments for breast cancer [3,4,5]. Studies have also shown that these physical changes can lead to a decreased level of physical functioning and have a negative impact on the quality of life (QOL) of the individual [6,7,8,9].

The detrimental effects of cancer and cancer related treatment on the physical function and QOL of BCS vary in magnitude and longevity [10]. Ganz *et al*. (2004) reported that BCS experience significant decrements in their perceived physical function at the cessation of their primary treatment for breast cancer [11]. Unfortunately, there has also been research that shows these negative effects on QOL and physical function extend far into survivorship, even though the primary treatments for cancer have been long finished [12]. Specifically, Simonavice *et al*. (2011) found that 17 months after the completion of primary treatment for breast cancer, BCS exhibited 21% lower strength for chest press and 23% lower strength for leg extension compared to age-matched healthy physically inactive women. Similarly, in this same study the authors found that BCS reported an 11% lower subjective physical function as measured via the Short Form-36 Health Survey (SF-36) and demonstrated lower objective physical function scores that were approaching significance compared to age-matched healthy physically inactive controls [13]. The fact that individuals have conquered breast cancer only to remain suffering from decreased physical abilities and QOL warrants the implementation of interventions aimed to correct these deficits.

Over the past several years, researchers have investigated the effects of various exercise modalities and intensities on QOL in cancer patients and survivors. According to a recent meta-analysis, there are numerous studies that have indicated resistance training (RT) interventions can successfully improve QOL among cancer patients and survivors [14]. Despite these findings, there are also reports that indicate resistance exercise interventions fail to elicit any positive QOL changes in cancer patients and survivors [15,16]. This inconsistency in the research warrants further investigation to examine the effects of resistance exercise on QOL in cancer patients and survivors. Furthermore, of the studies examined, physical function is often measured from subcategories within QOL questionnaires (*i.e*., Short Form Health Survey and Functional Assessment of Cancer Therapy—General). To date there has only been one study examining the efficacy of resistance training exercise to increase objective physical function in cancer survivors. Jankowski *et al*. (2008) implemented the Continuous Scale Physical Functional Performance (CS-PFP) test in a group of older cancer survivors and found that components of the CS-PFP were significantly higher compared to the control group after a resistance training exercise intervention [17]. The void in literature examining the efficacy of resistance training to increase the objective physical function of cancer patients and survivors demonstrates the need for further research utilizing objective assessments of physical function. Furthermore, the CS-PFP test has been utilized as a tool to establish thresholds for independent living in the elderly population [18]. Obtaining a CS-PFP score for cancer survivors could possibly provide the information necessary to ensure that they maintain an adequate physical function capacity that will allow them to maintain their independent living status.

Studies reporting the negative physical and psychological changes that BCS encounter, and the lack of studies investigating non-pharmacological approaches to combat these negative changes, warrant an investigation of interventions to improve the conditions of this population. Thus, the purpose of the present investigation was to determine the efficacy of resistance exercise training on improving physical function, measured objectively, and subjective physical function and QOL in BCS. It was hypothesized that BCS would demonstrate improvements in both objective and subjective physical function as well as QOL in response to a six-month resistance training program.

## 2. Experimental Section

### 2.1. Participants

Female BCS (stages 0-III), having completed treatments at least six months prior to beginning the study were recruited. Participants were recruited via advertisements posted at various local establishments and announcements made at local breast cancer support groups. Participants currently taking or who had completed hormone suppressant therapies were eligible to participate in this study. Participants still receiving hormone suppressant therapies were eligible for the study because after initial treatments are completed, hormone suppressant therapies are typically prescribed for an additional period of three to five years. Excluding women still taking hormone suppressant therapies would have significantly decreased the number of women eligible for the study. Participants already participating in vigorous exercise programs at baseline of the study and/or those with uncontrolled hypertension (≥160/≥100 mmHg), uncontrolled diabetes, uncontrolled heart disease, or who were still going through cancer treatment were not allowed to participate in the study.

### 2.2. Procedures

Prior to baseline assessments, participants were given a physician consent form to take to their primary health care providers to be evaluated for participation in the study. Upon clearance by their physician, participants were provided an opportunity to ask any questions about the research study requirements or expectations. After all questions and concerns had been addressed, participants completed a written informed consent document. Participants then completed a demographic and medical history questionnaire and were given an appointment for baseline assessment. This study was approved by the Institutional Review Board at The Florida State University.

### 2.3. Data Collection

At their scheduled appointment, participants arrived to the testing laboratory for their first baseline appointment, at which point participants completed the SF-36 for a baseline QOL assessment of physical function, and both physical and mental well-being. Following the questionnaire, the participants had their resting blood pressure, resting heart rate taken and their upper and lower body strength measured. Upper and lower body strength were assessed using the chest press and leg extension exercises, respectively (MedX™, Orlando, FL, USA). After a warm-up, participants were progressed towards the maximum weight that they could lift one time through a full range of motion to achieve a one repetition maximum (1RM). The 1RM tests for both upper and lower body were obtained within a 10–15 min time frame after the initial warm-up set and were performed according the guidelines for strength testing as outlined by the American College of Sports Medicine [19].

On the second baseline visit, which occurred one week later, participants had their physical function measured objectively via the CS-PFP test. The CS-PFP test was developed using data collected on older adults with a broad range of physical abilities [20]. This test has been shown to have convergent, construct, and face validity for 10 everyday household tasks. It has high reproducibility (*r* = 0.97) and is sensitive to change, induced by exercise, with an effect size of 0.8 [20,21]. The CS-PFP is specific for physical function and is not related to emotional or mental health or depression [21]. The CS-PFP test has also been identified as having the ability to predict living dependency status from the threshold score of 57 units [18].

The CS-PFP is based on routine tasks, performed at maximal effort within the bounds of safety and comfort. A total of 10 tasks are administered, and a combination of time, distance, and weight is used to quantify performance. Tasks quantified using both weight and time include: (1) carrying of weight and (2) carrying groceries. Tasks quantified by time alone include: (1) transferring laundry from a washer to a dryer, (2) putting on and removing a jacket, (3) floor sweeping, (4) climbing stairs, (5) getting down and up from the floor, and (6) picking up four scarves from the floor. Tasks that are quantified by distance alone include: (1) a six-minute walk and (2) highest reach. Each task is scored 0–100, based on an empirically derived range from data gathered on older adults with a broad range of individual functional abilities [20].

Time was used to calculate speed (1/t), so that higher numbers reflected higher function for each unit of measure (weight, distance, and speed). Each task is scaled 1–100 according to the following formula:

Corrected Score = (observed score – lower limit)/(upper limit – lower limit) × 100

The total physical functional performance score (CS-PFP total) is the average corrected score of all tasks. The CS-PFP total can also be broken down into five domains representing upper body strength, upper body flexibility, lower body strength, balance and coordination, and endurance.

The laboratory for the administration of the CS-PFP test was set up to adhere to the published dimensions [20] and was administered using the published protocol [21] and a scripted dialog with minor changes tailored to the laboratory at which the present study was conducted. Before the start of the CS-PFP test, all women had the procedures for testing explained to them. They were told to “perform each task safely, working as fast as you can”. At the completion of testing participants were asked to rate their perceived effort (RPE) for the entire testing procedures of the CS-PFP test from 6 to 20 on the Borg scale [19]. Heart rate was monitored continuously during the test. After the completion of the CS-PFP, maximal strength tests were verified by repeating the 1RM as outlined in day one of baseline testing. The highest measurement for the upper and lower body from the two days of testing was considered the 1RM and used for calculating the resistance training exercise prescription. All baseline measurements were repeated at three and six months.

### 2.4. Intervention

After the completion of baseline testing, each participant was provided an appointment for their supervised twice weekly resistance training sessions. For each exercise session, participants were prescribed a target goal of two sets of 8–12 repetitions at 60%–80% of their 1RM. Exercise machines included the MedX^TM^ chest press, leg press, leg extension, biceps curl, triceps press down, overhead press, seated row, leg curl, abdominal crunch, and lower back hyperextensions. During each resistance exercise session, participants performed a warm-up by walking for approximately five minutes, and concluded their session by performing stretches that targeted all the major muscle groups. Total exercise session time was approximately one hour. Exercise intensities for the chest press and leg extension were calculated as a representation of upper body and lower body intensity, respectively. When assessing the intensity (percentage of 1RM) for a particular four-week period, the 1RM test just prior to the four-week period was used for calculating percentage of 1RM lifted. A complete account of the resistance training intervention is published elsewhere [22].

### 2.5. Statistical Analysis

A time effect, with an effect size of 0.85, maintaining α = 0.05 and 1-β = 0.80, indicated that a sample size of at least 10 participants was required to detect changes in total CS-PFP scores over time based on a previous finding by Ades and colleagues [23]. Sample size calculation was found by using G*Power 3 software [24]. All analyses were performed using the SPSS (version 22, IBM Corp., Armonk, NY, USA) statistical package. Descriptive statistics (means, standard deviations) were calculated for all variables. Dependent variables, CS-PFP scores, SF-36 scores, muscular strength, and participant characteristics were analyzed by repeated measures analysis of variance (ANOVA) with repeated measures performed on the time factor (baseline, three months, and six months). In the case of significant findings from the ANOVA, main effects were compared using a Bonferroni post hoc test. In cases of sphericity violations, Greenhouse-Geisser adjustment was used to test time effects on the dependent variables. Pearson Product Moment Correlations were utilized to assess significant relationships between the dependent variables of muscular strength, SF-36, and CS-PFP. An intention to treat analysis was implemented for all subjects that were unable to complete the intervention; therefore, a three-month assessment time point was implemented into the research project in the case a participant ceased participation from three months to six months. All significance was accepted at *p* ≤ 0.05. All data are presented as means ± standard deviations.

## 3. Results and Discussion

### 3.1. Participant Characteristics

A total of 51 BCS potential participants inquired about the study; 10 declined participation after initial screening; and nine did not meet qualifying criteria. Thirty two participants completed baseline testing; however five women did not return after initial testing. Two women were unable to complete the RT invention due to medical complications that surfaced during the course of the study and stopped all participation in the research project. One participant developed uncontrolled hypertension during the 18th week of the study and her physician would not grant clearance to remain in the study. The second participant, during the 8th week of the study was diagnosed with a reoccurrence of cancer that metastasized into her bones and was not permitted to continue with the study. Since an intention to treat analysis was implemented a total of 27 participants were utilized to carry out the research project.

Analysis of the participants’ baseline characteristics revealed the women were 64 ± 7 years of age, weighed 73.5 ± 14.8 kg, and had a height of 163.1 ± 6.1 cm. Table 1 provides physical baseline characteristics of the participants. The majority, 87% (*n* = 20) of the participants were Caucasian, while the remaining 13% (*n* = 3) were African American. Table 2 provides details of the cancer diagnosis and treatment histories of the participants.

**Table 1:** Participant Characteristics (*N* = 27).

Variable	M ± SD	Range
Age (years)	64 ± 7	51–74
Body weight (kg)	73.5 ± 14.8	53.7–106.6
Height (cm)	163.1 ± 6.1	151–179
Body mass index (kg/m^2^)	27.7 ± 5.5	21.8–41.2

**Table 2:** Participant Cancer Related Characteristics (*N* = 27).

Frequency breast cancer stage and breast affected	
Stage 1	10
Stage 2	13
Stage 3	4
Affected breast—left	9
Affected breast—right	18
**Time since diagnosis and treatments**	
Time since diagnosis (months)	87.9 ± 68.4
Time since hormone therapy completed (months)	53.6 ± 43.1
Time since *primary treatment completed (months)	75.9 ± 65.6

Data are presented as mean ± standard deviation, * Surgery, radiation, and/or chemotherapy.

### 3.2. Muscular Strength

The participants displayed a steady progression of weight lifted for upper and lower body exercises throughout the six-month intervention. For weeks 1–4, participants exercised at an intensity less than the target intensity (60%–80% 1RM); with the participants achieving an intensity of 52% ± 9% 1RM for upper body and 52% ± 5% 1RM for lower body. Beginning in weeks 5–8, participants achieved an exercise intensity of >60% 1RM for both upper and lower body and continued to maintain compliance to the study design of >60% 1RM for the remaining weeks of the intervention. At no point in the study did the participants exercise over 69% of their previous four-week 1RM for upper or lower body. Throughout the intervention, the participants were able to significantly increase their upper and lower body strength. There were significant time effects observed for chest press strength (*F*_(1.326, 25)_ = 32.913, *p* ≤ 0.01, *ES* = 0.568) and leg extension strength (*F*_(1.307, 25)_ = 51.073, *p* ≤ 0.01, *ES* = 0.671). There was a significant 17% increase in upper body strength from baseline to three months and a further significant increase of 7% from three months to six months, for an overall 25% improvement from baseline to six months. Similarly, there was a significant 15% increase in upper body strength from baseline to three months and a further significant increase of 9% from three months to six months, for an overall 26% improvement from baseline to six months. Table 3 provides a complete description of the strength variables. A complete account of the resistance training intervention results and progression is published elsewhere [22].

**Table 3:** Muscular Strength (*N* = 27).

Variable	Baseline	3-month	6-month
1RM Chest Press (kg)	71 ± 22	83 ± 21**^a^**	89 ± 22**^a, b^**
1RM Leg Extension (kg)	74 ± 18	85 ± 23**^a^**	93 ± 24**^a, b^**

Data are presented as mean ± standard deviation; 1RM=1 repetition maximum. **^a^** Significantly different from baseline, p ≤ 0.05; **^b^** Significantly different from 3-month, p ≤ 0.05.

### 3.3. CS-PFP

There were significant time effects observed for the subcomponents of total function of upper body strength component (*F*_(2, 25)_ = 12.457, *p* ≤ 0.05, *ES* = 0.324), upper body flexibility (*F*_(2, 25)_ = 3.452, *p* ≤ 0.05, *ES* = 0.117), lower body strength component (*F*_(2, 25)_ = 25.817, *p* ≤ 0.05, *ES* = 0.498), balance and coordination component (*F*_(2, 25)_ = 18.613, *p* ≤ 0.05, *ES* = 0.417), endurance component (*F*_(2, 25)_ = 19.569, *p* ≤ 0.05, *ES* = 0.429), and total function (*F*_(2, 25)_ = 23.896, *p* ≤ 0.05, *ES* = 0.479). From baseline to six months, the participants experienced significant improvements in their upper body strength (+12%), lower body strength (+17%), balance and coordination component (+12%), endurance component (+12%), and total function (+12%). Scores at baseline for total function ranged from 36 to 82 units; with six participants achieving a score that fell below the threshold score of 57 units, which is needed for independent living. At the six-month mark, the scores for total function ranged from 50 to 93 units, with two participants remaining at a score that fell below the threshold score of 57 units. Despite the significant ANOVA for upper body flexibility, examination of the main effects failed to show any significant changes among any of the three time points. Table 4 provides a complete description of the CS-PFP variables.

**Table 4:** Comparison of Continuous Scale-Physical Functional Performance* (*N* = 27).

Variable	Baseline	3-month	6-month
Upper body Strength (units)	63.5 ±16.3	68.9 ± 15.15 ^a^	71.2 ± 16.8 ^a^
Upper body Flexibility (units)	81.1 ± 8.2	82.9 ± 9.3	84.0 ± 6.4
Lower body Strength (units)	58.5 ± 14.9	64.5 ± 15.2 ^a^	68.6 ± 16.3 ^a,b^
Balance & Coordination (units)	66.5 ± 12.2	71.8 ± 12.1 ^a^	74.6 ± 11.6 ^a^
Endurance (units)	67.2 ± 12.0	72.4 ± 11.9 ^a^	75.0 ± 11.6 ^a^
Total function (units)	65.5 ± 12.1	70.7 ± 12.2 ^a^	73.6 ± 12.2 ^a,b^

* Scores range from 0 to 100; 0 = worst function; 100 = best function; Data are presented as mean ± standard deviation; ^a^ Significantly different from baseline, *p* ≤ 0.05; ^b^ Significantly different from 3-month, *p* ≤ 0.05.

### 3.4. SF-36

Participants’ physical function, mental QOL, and the physical QOL components of the SF-36 Health Survey were unchanged at any of the of the time points. See Table 5 for a complete description of the components of the SF-36 Health Survey.

**Table 5:** Comparison of Short Form (36) Health Survey * (*N* = 27).

Variable	Baseline	3-month	6-month
Physical function	82.6 ± 13.6	83.9 ± 14.8	84.6 ± 16.2
Mental QOL	52.0 ± 10.3	50.8 ± 12.1	52.5 ± 10.9
Physical QOL	49.0 ±7.1	49.2 ± 8.2	48.3 ± 9.9

* Scores range from 0 to 100; 0 = worst; 100 = best; Data are presented as mean ± standard deviation; QOL = Quality of life.

### 3.5. Correlation between Strength and CS-PFP

There were several significant correlations between percent change in upper body strength and percent change in several components of the CS-PFP test. Specifically, percent change of upper body strength was significantly related to percent change in total CS-PFP function (*r* = 0.39, *p* = 0.05). Similarly, there were several significant correlations between percent change in lower body strength and percent change in several components of the CS-PFP test. Specifically, percent change of lower body strength was significantly related to percent change in total CS-PFP function (*r* = 0.41, *p* = 0.04). There were no significant correlations found between percent change in CS-PFP and percent change in SF-36. A complete list of correlations between percent change in CS-PFP and percent changes in upper body and lower body strength and SF-36 test can be found in Table 6.

**Table 6:** Pearson Product Moment Correlations between Percent Change in CS-PFP* and Percent Changes in Strength and SF-36** (*N* = 27).

Variable	CS-PFP Upper Body Strength	CS-PFP Lower Body Strength	CS-PFP Upper Body Flexibility	CS-PFP Endurance	CS-PFP Balance and Coordination	CS-PFP Total Function
Upper Body Strength	*r* = 0.08, *p* = 0.70	*r* = 0.34, *p =* 0.87	*r* = 0.43 ^a^, *p =* 0.03	*r* = 0.42 ^a^, *p =* 0.03	*r* = 0.15, *p =* 0.45	*r* = 0.39 ^a,^ *p =* 0.05
Lower Body Strength	*r* = 0.22, *p =* 0.27	*r* = 0.47 ^a^, *p =* 0.02	*r* = 0.08, *p =* 0.70	*r* = 0.39 ^a^, *p =* 0.05	*r* = 0.36, *p =* 0.07	*r* = 0.41 ^a^, *p =* 0.04
SF-36 Physical Function	*r* = −0.03, *p =* 0.88	*r* = 0.06, *p =* 0.75	*r* = −0.11, *p =* 0.58	*r* = 0.07, *p =* 0.72	*r* = 0.01, *p =* 0.95	*r* = 0.03, *p =* 0.90
SF-36 Mental Quality of Life	*r* = −0.14, *p =* 0.49	*r* = −0.01, *p =* 0.96	*r* = −0.16, *p =* 0.42	*r* = −0.04, *p =* 0.84	*r* = 0.09, *p =* 0.64	*r* = −0.06, *p =* 0.77
SF-36 Physical Quality of Life	*r* = 0.18, *p =* 0.37	*r* = 0.16, *p =* 0.42	*r* = 0.18, *p =* 0.37	*r* = 0.16, *p =* 0.44	*r* = 0.03, *p =* 0.87	*r* = 0.15, *p =* 0.47

*: CS-PFP = Continuous Scale-Physical Functional Performance; **: SF-36 = Short Form (36) Health Survey; ^a^: Correlation is significant *p ≤* 0.05.

## 4. Conclusions

The present study investigated the efficacy of resistance training in improving the physical function and QOL in a sample of BCS. All of the women had high adherence to the resistance training sessions (96%). The women demonstrated significant improvements in total function and all subcomponents of the CS-PFP, thus the hypothesis that a resistance training intervention would improve objection function in BCS was supported. No changes were reported for subjective levels of physical or mental QOL, measured via the SF-36 Health Survey; thus the hypothesis that a resistance training intervention would improve QOL and subjection function in BCS was rejected.

Women participating in the study showed excellent capabilities to improve objective physical function; however, the same cannot be said for the subjective levels of physical function or for QOL. To date the present study was the first to implement the CS-PFP test among BCS for an assessment of objective physical function. Results showed that the participants increased total function by 12%. While there are no studies examining BCS to which the results of the present study can be compared, Jankowski *et al*. (2008) implemented the CS-PFP in a group of older cancer survivors (cancer type not specified) and found that after four months of RT, both upper body and lower body strength components of the CS-PFP were significantly higher as compared to the control group [17]. Total physical function was not accounted for by Jankowski *et al.* (2008). The baseline values for the women from the present study were seemingly higher for the upper body strength component (63.5 ± 16.3 units) as compared to the baseline values from Jankowski and colleagues (59 ± 29 units). Similarly, for the lower body strength component, the women from the present study had higher baseline values (58.5 ± 14.9 units) compared to Jankowski and colleagues (45 ± 16 units). These discrepancies are likely due to the older population (71 ± 5 years) with which Jankowski and colleagues studied, as compared to the present study where the sample population was younger (64 ± 7 years). Although time effects were not reported by Jankowski *et al*. (2008), pre-to-post differences were calculated to be +20% for the upper body strength component and +11% for the lower body strength component [17]. These improvements are similar to the results of the present study. Another study reported that a resistance training intervention significantly improved six-minute walking distance in a sample of BCS [25]. The six-minute walk test is essentially the “endurance” component of the CS-PFP. Thus, these results are in agreement with the 12% improvement in the endurance component of the CS-PFP test as seen respectively for the participants of the present study. While there is no minimum clinically importance difference established for the CS-PFP test, the improvements in CS-PFP from the present study were significantly correlated with improvements in the participants’ upper and lower body strength, as measured by 1RM assessments.

Although, there is a lack of literature in which to compare the CS-PFP results of the present study with cancer survivor populations, the results found align with published data in other female chronic diseased populations. Kingsley and colleagues (2005) reported that 12 weeks of resistance training in women with fibromyalgia, resulted in a 14% increase in total CS-PFP function [26]. Whereas, Brochu *et al*. (2002) reported an even larger improvement (+24%) in total function in a group of older, disabled women with coronary heart disease after six months of resistance training [27]. These results in combination with the +12% increase in total CS-PFP function experienced in the present study tout the effectiveness of resistance training to improve objective physical function.

The ability for BCS to increase physical function is especially important given the fact that after the completion of cancer treatments, BCS have significantly (*p* = 0.08) lower physical function scores as compared to healthy age-matched women who were physically inactive [9]. It should also be noted that while the baseline values from the present study for total function (65.5 ± 12.1 units) mimicked those of the BCS baseline values for total function (66.1 ± 13.8 units) from Simonavice and colleagues, the six-month values from the present study for total function (73.6 ± 12.2 units) more closely mirrored the baseline results from the healthy controls (75.1 ± 13.0 units) from Simonavice and colleagues [9]. These results imply that resistance training is an effective way to improve the physical functional status of BCS to that of healthy women. Additionally, it should be highlighted that in the present study at baseline, six participants achieved a CS-PFP score that fell below the threshold score of 57 units, which is needed for independent living [18]. At the six-month mark, only two participants had a score that remained below the threshold score of 57 units, indicating that the intervention was able to bring four BCS above the functional threshold needed for successful independent living. The two BCS that remained under the 57 unit functional threshold were still able to make improvements from baseline to three months and then able to maintain their heightened score from three months to six months. Specifically, the two BCS scored 36 units and 45 units at baseline and were both able to increase their scores to 50 units by three months and maintain a score of 50 at six months, demonstrating an 11% and 39% increase in total function, respectively.

The significant correlations that were found between both upper and lower body strength and total CS-PFP function are similar to that found in previous studies investigating strength and function. While the present study is unique in that CS-PFP was used to assess objective function in BCS, previous studies have assessed function using other modalities in older adult populations. Fukagawa *et al*. (1995) found that lower body strength was significantly related to lower body function as measured with chair stands in a group of elderly individuals [28]. These results are similar to those found with Chandler *et al*. (1998) who found that lower extremity strength was significantly related to chair stand performance and gait speed in older adults [29]. While the relationship between upper body strength and function is less clearly defined in past literature compared to lower body strength, Foldvari *et al*. (2000) found a significant relationship between upper body strength and subjective self-reported function in elderly women [30]. These findings are consistent with the results of the present study which indicated a significant relationship between upper body strength and function as measured by total CS-PFP. The relationship between upper body strength and function is especially important in BCS because of the high incidences of upper body morbidities that accompany a breast cancer diagnosis. Specifically, Hayes *et al*. (2012) state that 10%–64% of BCS report upper body morbidities from six months to three years after diagnosis [31]. These morbidities in combination with the severity of the cancer and cancer-related treatments dispose BCS to a loss of upper body function [32]. The results of the present study are encouraging in that if BCS can improve their upper body strength during recovery from a breast cancer diagnosis, it is likely that physical function will also be favorably affected.

The lack of QOL improvement for the BCS in the present study was inconsistent with most previous literature investigating QOL changes in cancer survivors after an exercise intervention. Many studies have reported that following resistance training interventions of various durations, intensities, and volumes have produced significant improvements in QOL in BCS [33,34,35]. The difference between these previous studies and the present study is the type of subjective questionnaire utilized. The Functional Assessment of Cancer Therapy-General (FACT-G) or the Functional Assessment of Cancer Therapy-Breast (FACT-B) were the most commonly used survey tools assessing QOL among the studies reviewed. The present study implemented the Short Form-36 Health Survey (SF-36). The lack of significant improvement for QOL in the present study suggests that the FACT-B and FACT-G may address more specific questions regarding the impact that cancer and cancer-related treatments may have on QOL and thus may be more sensitive to detecting QOL changes within BCS. Despite the inconsistencies of the present QOL results with those previously published in cancer survivors populations, the lack of significant findings for improvement in the SF-36 QOL components in the present study are similar to that found in various studies with older and chronic diseased populations. Ades and Meyers (2003) reported no improvements in the physical function QOL component of the SF-36 after a six-month resistance training intervention with older female cardiac patients [23]. Similarly, Barrett and Smerdely (2002) reported no significant improvement in the physical function QOL component of the SF-36 after a ten-week resistance training intervention with older individuals [36]. The baseline scores from the participants of the present study for physical function QOL component were 82.6 ± 13.6, which are notably higher than previous studies, such as Ades and Meyers, who reported a baseline score of 59 ± 20 or Barrett and Smerdely, who reported baseline scores of 71 ± 18. The higher baseline scores for the SF-36 components reported in the present study could have increased the difficulty to detect significant changes over time. Despite the fact that neither the physical nor mental QOL scores were changed over the course of the six-month intervention, the fact remains that all the BCS from the present study significantly improved physical function, as measured objectively via the CS-PFP. Also noteworthy is the fact the present study failed to show any significant correlations between the percent changes of function measured subjectively via the SF-36 and objectively measured via the CS-PFP. These results emphasize the importance of objective measures of physical function in the BCS population.

The present study had several limitations that may have hindered the ability to accurately interpret the results. The present study had a seemingly smaller sample size than other studies of similar design, which may have hindered the obtainment of statistical significance for some of the variables assessed. Another limitation of the current study was a lack of a true control group, which may have lessened the magnitude of the results reported. Lastly, the usage of the SF-36 QOL instrument may not have been sensitive enough to accurately represent the physical and mental changes that BCS experienced over the course of the intervention. In fact, previous literature suggests that self-reported questionnaires, such as the SF-36, are not sensitive enough to allow individuals to accurately report subtle changes in physical function that may be clinically relevant [23].

In conclusion, our findings indicate that a resistance training intervention of moderate intensity was well tolerated among BCS. All women displayed high levels of adherence to the attendance of exercise sessions and reported no adverse physical incidences as a result of the intervention. With the exception of upper body flexibility, the women improved all other subcomponents, as well as total function, measured via the CS-PFP. It is also noteworthy that at the end of the six-month intervention, upper and lower body strength as well as objective physical function measures were increased to levels that mimicked those achieved by healthy inactive post-menopausal women. This implies that a resistance training intervention is capable of helping BCS achieve similar levels of strength and function that they may have had prior to their diagnosis and treatment of breast cancer. Also noteworthy is the fact that the participants of the present study were able to make the aforementioned gains from a light to moderate intensity resistance training intervention (52%–69% 1RM). The efficacy of a light to moderate intensity resistance training program should be emphasized among health care practitioners and can possibly motivate cancer survivors to begin a more conservative resistance training program who are hesitant to begin strenuous activity. The present study was unable to detect any changes in QOL among the participants; however, from the literature reviewed, the QOL assessment tool may have not been the best choice for the BCS population. Furthermore, the significant gains in objective levels of physical function that the participants achieved should be more heavily considered as opposed to the subjective assessment of QOL, as it provides a more accurate depiction of their true physical capacities and physical well-being.
